# Inhibiting KMO blocks kynurenine metabolites’ induction of senescence in human neurons, microglia, and 5xFAD mice

**DOI:** 10.1093/geroni/igaf122.3967

**Published:** 2025-12-31

**Authors:** William Hill, Dmitry Kondrikov, Steve Dixon, Gavin Wang

**Affiliations:** Medical University of South Carolina, Charleston, South Carolina, United States; Medical University of South Carolina, Charleston, South Carolina, United States; Medical University of South Carolina, Charleston, South Carolina, United States; Medical University of South Carolina, Charleston, South Carolina, United States

## Abstract

There is a growing interest in the involvement Kynurenine (KYN) Pathway (KP) metabolites in neurodegenerative disorders like Alzheimer’s and Parkinson’s disease. Research in this area is still limited but shows potential for providing novel mechanisms of action to target Alzheimer’s disease therapeutically. Neuroinflammatory factors like INF-g, TNF-a, and IL-6 upregulate IDO-1 conversion of the essential amino acid tryptophan into KYN. In turn kynurenine monooxygenase (KMO) modifies KYN into 3-hydroxykynurenine (3-HK). Importantly, we have demonstrated that downstream KYN metabolites 3-HK, 3-Hydroxyanthranilic Acid (3-HAA), and quinolinic acid (QA) act in part via the Aryl hydrocarbon receptor (AhR), signaling system to induce senescence, which can be blocked by AhR inhibition (siRNA and DFM). Our preliminary data demonstrated that 3-HK,3HAA, and QA induced senescence in human neurons (SH-SY5Y cells) and microglial cells (HMC3), by multiple assays (including p21, SAb-Gal, H3K9Me3 methylation nuclear staining, and DNA damage), in a dose dependent fashion, as well as generating reactive oxygen species (ROS).These metabolites also increased tau phosphorylation in SH-SY5Y cells. Inhibiting KMO with siRNA, JM6, R0-61-8048 or GSK366 reduces generation of sub-KYN metabolites and decreases their ability to induce senescence or generate inflammatory SASPs and ROS. We treated 3-month-old 5xFAD Alzheimer’s disease model mice with 3-HK or with the KMO inhibitor JM6 for 2 months. We saw an increase in senescence biomarkers with 3-HK (PAI-1, p21, IL-6, TIMP2) relative to a decrease with JM6 KMO inhibition. Our studies support exploring the targeting of KMO to potentially reduce central nervous system senescence.

